# Do mouthwashes affect the optical properties of resin cement?

**DOI:** 10.1186/s12903-024-04044-9

**Published:** 2024-02-25

**Authors:** Nazire Nurdan Çakır Kılınç, Pınar Yıldız

**Affiliations:** 1 Nuh Naci Yazgan University, Faculty of Dentistry, Department of Restorative Dentistry, Kayseri, Turkey; 2 Nimet Bayraktar Oral and Dental Health Center, Kayseri, Turkey

**Keywords:** Colour stability, Contrast ratio, Mouthwash, Resin cement, Translucent parameter

## Abstract

**Objectives:**

The objective of this study was to evaluate the effect of mouthwashes on the optical properties of resin cement.

**Materials and methods:**

One hundred and 60 resin cement discs (6x2mm) were produced from 4 different brands of resin cement (Panavia V5, Estecem II, RelyX Veneer, NX3) with the help of a Teflon mould. The discs were divided into 4 subgroups, 1 of which served as the control group, to be immersed in mouthwashes after measuring the initial L, a, and b values on white and black backgrounds. Colour measurements were repeated after the 1st and 7th days. The collected data were used to calculate the ∆E_00_ value to measure colour stability, the translucency parameter (TP_00_), and the contrast ratio parameter (CR) to compare translucency change. Data were statistically analysed using mixed-design analysis of ANOVA and the Bonferroni-Dunn test. Repeated measures ANOVA was used for dependent results (α = 0.05).

**Results:**

On the ∆E_00_, TP_00_, and CR parameters; the joint effect of resin groups, mouthwash groups, and measurement times were found to be statistically significant. The ∆E_00_ (colour difference) parameter; the joint effect of resin groups, mouthwash groups, and measurement times was found to be statistically significant. The TP_00_; the joint effect of resin groups, mouthwash groups, and measurement times were found to be statistically significant. The CR parameter; the joint effect of resin groups, mouthwash groups, and measurement times was found to be statistically significant. In the Estecem II (Tokuyama) group, the means of Listerine Cool Mint (Johnson & Johnson) was above acceptable limits in both time periods. In the Panavia V5 (Kuraray Noritake) group, the color change was above acceptable limits in all time periods and in all mouthwash groups. Among the resin materials used, Estecem II (Tokuyama) shows the most color change. Listerine Cool Mint (Johnson & Johnson) caused more color change than other mouthwashes at all times.

**Conclusions:**

Within the limitations of this study; the colour stability and translucency value of resin cement depend on both the resin cement content and the mouthwash. Long-term use of mouthwash may adversely affect the optical properties of the resin cement.

**Clinical relevance:**

Clinicians should take into account the fact that mouthwash use and the composition of the resin cement employed will have an impact on the colour of laminate veneers.

## Introduction

Materials used in dentistry should be able to reflect the optical properties of natural dentin and enamel tissue. All-ceramic restorations can provide natural-looking rehabilitation when used correctly [1].

With the developments in ceramic technology in laminate veneers, which provide more conservative tooth preparation, thinner and more translucent restorations can be produced. With existing ceramic materials, the thickness of laminate veneers can vary between 0.3 and 0.7 mm. Factors that affect the aesthetics of veneer restorations include the underlying tooth structure, the translucency, and opacity of the ceramic material used, the colour of the luting cement, and the colour of the veneer [1].

Although there is no consensus in the literature regarding the colour stability of the final restoration, the colour and translucency of the resin cement used affect the final colour of the laminate veneer after cementation [2].

Translucency is one of the important factors in ensuring the colour equality of natural teeth with restorative materials [3]. Translucency is defined as “the property of a material in which a large part of the transmitted light is scattered” [3]. Additionally, translucency can be defined as a state of partial opacity, or between full opacity and full transparency, where the material allows the appearance of the background as a result of the passage of light [4]. Higher values for TP_00_ represent greater translucency of the material; if the material is completely opaque, the value of this parameter is zero [5]. Translucency in aesthetic restorative materials affects colour harmony with neighbouring or adjacent teeth or restorations as well as the depth of colour in restorations. Commission Internationale de L’Eclairage (CIE) colour coordinates and CIE standard illuminants are frequently employed for optical property measurements [6]. Two indices, such as the translucency parameter (TP_00_) and contrast ratio (CR), have been frequently employed to determine translucency [7, 8].

Translucency impacts colour-matching with neighbouring or adjacent teeth or restorations as well as the depth of colour in restorations. Poor shade mixing of opaque restorative materials at the tooth interface may affect the optical performance of restorations [9].

Other optical properties evaluated in resin-based materials are opalescence and fluorescence parameters. When light reflects off the restorative material, it should appear blueish, and when light passes through it, it should appear orange. Opalescence is a phenomenon that is crucial for accurately replicating the actual structure of enamel. The opalescence parameter (OP) controls it [10]. Natural teeth seem whiter and brighter in daylight because they glow blue under ultraviolet (UV) light. The emission of light by healthy teeth that have absorbed light is known as fluorescence. Using a spectrophotometer, it is possible to evaluate whether dental materials are fluorescent by comparing their color in the presence and absence of UV radiation. This data is called the fluorescence parameter [11].

For a long time ensuring colour harmony and aesthetics in restoration and maintaining this harmony has been one of the most important success criterion. The perceptible discolouration of resin-based materials can cause patient dissatisfaction and the need to renew the restoration, thus resulting in additional treatment costs.

Colour changes in resin-based materials are related to their chemical structure or to external factors to which they are exposed. The colouring of resin-based materials depends on many factors, including intrinsic and the extrinsic colouration factors. In term of internal colour changes; there is a correlation between colour stability and the degree of conversion. The oxidation of unreacted amines from the aromatic tertiary amines involved in the curing reaction of polymerized materials is the main cause of the internal colouration of the restoration. These amines are used in large amounts in order to chemically cure resin-based materials while light-curing resin-based materials are in smaller amounts. However, incompletely polymerized resin-based materials are more susceptible to colouration. With regard to water solubility and water absorption; they cause swelling, plasticization and softening oxidation, and hydrolysis of the resin matrix. As a result, colour stability decreases, and sensitivity to colouration increases [12, 13].

External colour changes occur either on the surface or at the edges of the restoration and may occur as a result of insufficient polymerization, incorrect finishing and polishing, poor oral hygiene, and consumption of colouring substances such as tea, coffee, and cigarettes [14–16].

Mouthwashes contain water, antimicrobial agents, colouring agents, salts, preservatives, and sometimes alcohol in their structure and have different pHs according to the differences in their concentrations; In addition to preventing caries, they are widely used in terms of reducing bad breath, protecting periodontal health and providing a fresh breath [17]. However, they can have harmful effects on restorative materials depending on their pH as influenced by; active ingredients, and alcohol concentration. The low pH value of mouthwashes plays a key role in this mechanism [18].

It is stated that mouthwashes used for controlling intraoral infection and which provide antimicrobial activity may cause external discolouration of dental tissues and dental restorations [19]. Nowadays, with the increase in the use of very thin laminate veneers, the resin cement used has become very important in determining the final colour of the restoration, and colour stability may be affected by mouthwashes. In the literature, there are studies investigating the effects of mouthwash on resin-based materials, but no studies investigating the effects on resin cement discolouration have been found. Therefore, the aim of this study is to evaluate the effect of different mouthwashes on the optical properties of different types of resin cement. The initial hypothesis tested was that different mouthwashes would not make a difference in the colour stabilization and the optical properties of different types of resin cement.

## Materials and methods

This in vitro experimental study consisted of a consideration of 4 resin cement groups; (Panavia V5 (Kuraray Noritake, Okayama, Japan), Estecem II (Tokuyama, Dental Corp., Tokyo, Japan) RelyX veneer (3 M ESPE, St. Paul, USA), NX3 (Kerr, Corp., CA, USA) and 4 mouthwash groups 1 of which was the control group; (Control, Kloroben (Drogsan, Ankara, Turkey) Listerine Cool Mint (Johnson & Johnson, New Brunswick, N.J.) (Listerine CM), Listerine Total Care (Johnson & Johnson, New Brunswick, N.J.) (Listerine TC)). The materials used and their contents are shown in Table 1.

**Table 1:** Resin cements used in the study, polymerization type, color, composition and lot number

Resin cement	Manufacturer	Polymerization Type	Colour	Composition	Lot Number
Panavia v5	Kuraray Noritake, Okyama, Japan	Dual Cure	Clear	Bis-GMA, TEGDMA, Hydrophobic aromatic dimethacrylate, hydrophilic aliphatic dimethacrylate, Initiators, Accelerators, Silanated barium glass filler, Silanated fluoroaluminosilicate glass filler, Colloidal silica, Silanated aluminium oxide filler, dl Camphorquinone, Pigments	AQ0025
Estecem II	Tokuyama Dental Corp. Tokyo, Japan	Dual Cure	Universal	Bis-GMA, TEGDMA, Bis-MPEPP, silica-zirconia filler (74 wt%), camphorquinone	037E49
RelyX veneer	3 M ESPE, St.Paul, USA	Light Cure	A1-light yellow shade	Bis-GMA,TEGDMA, zirconia/silica filler.	NA28673
NX3	KERR CORP., CA,USA	Light Cure	Clear	Uncured methacrylate ester monomers, inert mineral fillers, activators and stabilizers, and radiopaque agent glycerine, water, fumed silica and inert glass powder, gelatin.	7,287,052

Bis-GMA, bis-phenol A diglycidylmethacrylate; TEGDMA, triethyleneglycoldimethacrylate; Bis-MPEPP, Bisphenol A polyethoxymethacrylate

### Specimen preparation

For the study, a total of 40 resin cement discs, 10 from each group, were obtained. The disc-shaped specimens were 6 mm in diameter and 2 mm thick. The samples were produced with the help of Teflon moulds placed between two sheets of glass. Dual polymerized resin cement was placed into Teflon moulds on the glass using their own mixing tips, while the light-cured resin cement was placed directly into the Teflon moulds on the glass and compressed between another sheet of glass placed on top of it. Light pressure was applied to the mould, which was compressed between two cement glasses, and a smooth surface was obtained by overflowing the excess material. All samples were polymerized for 20 seconds with an LED light device (Valo LED, Ultradent Products, South Jordan, USA) at a light intensity of 1000 mW / cm^2^. To standardize the distance between the sample and the light device, the light device was kept in contact with the glass placed on the samples. The prepared samples were kept in distilled water at 37 °C for 24 hours.

### Spectrophotometer measurements

The initial colour L, and, a, and b values of the samples were measured with the help of a spectrophotometer (Spectroshade Micro, MHT Optic Research AG, Goteborg, Switzerland). Measurements were made by a single researcher on a white background (L_w_, a_w_, b_w_) and a black background (L_b_, a_b_, b_b_) after the calibration of the device according to the company instructions, at a certain time of the day and in daylight. To prevent light from entering the samples from the environment, a silicone model compatible with the spectrophotometer was prepared around the samples. The model was placed on a flat surface. Measurements were made by touching the spectrophotometer to the model and positioning it vertically. Three L, a, and b values were measured from each sample’s entire surface area, and the average was accepted as the starting value. Afterward, the resin cement samples were kept in two different types of mouthwash Listerine Cool Mint (Johnson & Johnson) and, Listerine Total Care (Johnson & Johnson) and 1 antiseptic mouthwash (Kloroben, Drogsan) and distilled water for 1 day, each of which was prepared in 20 mL volume on the digital rotator (DSR-D-N1, Digisystem Laboratory Instuments Inc., New Taipei City, Taiwan). After the waiting process, the samples were washed with pressurized water for 3 minutes and removed from the solutions. Then, the colour measurements of the samples were then repeated as at the initial stage, and the colour change (ΔE_00_) values were calculated. The solutions of all samples were changed on a daily basis and the samples were kept in solutions for 7 days on the digital rotator. Continuous mouthwash exposure for 12 hours has been reported to be equivalent to 1 year of daily use (1 minute twice a day) by patients [20]. In this study, rinsing the samples in mouthwash for 1 day simulated 2 years of use, and rinsing in mouthwash for 7 days simulated 14 years of use. In this study, as stated by Paravina et al. [21], the clinically perceptibile/ acceptable limit was accepted as ΔE_00_ = 0.8/1.8.

Colour stability (ΔE_00_) were calculated according to the CIEDE2000 formulation as follows [22].

$$\Delta {E}_{00}=\sqrt[2]{{\left(\frac{\Delta {L}^{\prime }}{K_L{S}_L}\right)}^2+{\left(\frac{\Delta {C}^{\prime }}{K_C{S}_C}\right)}^2+{\left(\frac{\Delta {H}^{\prime }}{K_H{S}_H}\right)}^2+{R}_T\left(\frac{\Delta {C}^{\prime }}{K_C{S}_C}\right)\left(\frac{\Delta {H}^{\prime }}{K_H{S}_H}\right)}$$

with this formula, the CIELAB values were converted into CIEDE2000 L′, C′ (chroma), and h′ (hue) values. Based on the CIEDE2000 uniform colour space, ∆L’, ∆C′, and ∆H′ are estimated as metric differences between the samples’ corresponding values. To correct (weight) the metric differences to the CIEDE2000 differences for each coordinate, three empirical terms were used: KLSL, KCSC, and KHSH. RT stands for the rotation function, that is, the interaction between chroma and hue differences in the blue region. The parametric factors of the CIEDE2000 colour difference formula were set to in this study.

The translucency parameter (TP_00_) was calculated with the same formula from the data measured on white and black backgrounds [23].

The translucency parameter (TP_00_) was additionally calculated using the CIEDE2000 colour difference formula as follows:

$${TP}_{00}=\sqrt[2]{{\left(\frac{L{\prime}_B-L{\prime}_W}{K_L{S}_L}\right)}^2+{\left(\frac{C{\prime}_B-C{\prime}_W}{K_C{S}_C}\right)}^2+{\left(\frac{H{\prime}_B-H{\prime}_W}{K_H{S}_H}\right)}^2+{R}_T\left(\frac{C{\prime}_B-C{\prime}_W}{K_C{S}_C}\right)\left(\frac{H{\prime}_B-H{\prime}_W}{K_H{S}_H}\right)}$$

The subscripts “B” and “W” in this formula represent the specimens’ lightness (L’), chroma (C′), and hue (H′) over the black and white backgrounds, respectively. The rotation function (RT) explains how chroma and hue differences interact in the blue region. The parametric factors KL, KC, and KH are correction terms for the experimental conditions, while the weighting functions SL, SC, and SH adjust the total colour difference to account for variation in the placement of the colour difference specimen over the B and W backgrounds in L, a, and b coordinates. The parametric factors of the CIEDE2000 colour difference formula were set to 1 in this study. Discontinuities resulting from mean hue computation and hue-difference computation, both of which were identified and described by Sharma et al. [23], were taken into consideration when computing utilizing the CIEDE2000 colour difference formula.

The change in translucency that occurred at the end of the 1st day and the 7th day was calculated using the following formulaes.

$$\Delta {TP}_1={TP}_{00}1-{TP}_{00}0$$

$$\Delta {TP}_7={TP}_{00}7-{TP}_{00}0$$

In this study, the accepted perceptibility threshold was 0.62 and the acceptability threshold was 2.62 for ∆TP (translucency changing value) [24].

Lw and L_B_ coordinate values measured on white and black background were used to calculate the luminance as follows (Yn is equal to 100):

$$Y={\left(\frac{L+16}{116}\right)}^3x\ {Y}_n$$

The property known as CR assesses the transparency or opacity of materials by dividing the specimen’s reflectance against a black background by its reflectance against a white background [9]. The Contrast Ratio (CR) was calculated using the Y values of samples recorded on white (Yw) and black (Yb) backgrounds as follows:

$$CR=\frac{Y_B}{Y_W}$$

### Statistical analysis

The SPPS 25 (IBM Corp. Released 2017. IBM SPSS Statistics for Windows, Version 25.0. Armonk, NY: IBM Corp.) statistical package program was used to evaluate the data. The study was given descriptive statistics (mean, standard deviation, number, and percentile) for categorical and continuous variables. In addition, the homogeneity of the variances, one of the prerequisites of the parametric tests, was checked using the “Levene” test. The normality assumption was checked using the “Shapiro-Wilk” test. For repeated tests, the sphericity assumption was checked with the use of the “Mauchly” test and when the sphericity assumption was met, the “Sphericity Assumed” test was applied. For cases where the sphericity assumption was not met, the epsilon value was evaluated, and the “Huynh-Feldt” test was used for cases where it was greater than 0.75 and the “Greenhouse Geisser” test for cases where it was smaller was used for evaluation purposes. In our analysis, mixed-design analysis of variance and the “Bonferroni-Dunn” test, one of the multiple comparison tests, and the “Bonferroni-Dunn” test over time were used to make an overall assessment between resin groups, mouthwash groups, and repeated measurements. One-way ANOVA was used to compare more than two independent groups in one-way evaluations, and analysis of variance was used for repeated measures to compare more than two dependent groups. A significance level of α = 0.05 was considered.

## Result

When Table 2 is examined, the TP_00_ parameter, CR parameter, and ΔE_00_ (colour stability) parameter; and the joint effect of resin groups, mouthwash groups, and measurement times were found to be statistically significant. A large level effect was detected for the difference between the obtained means. It was seen that the 0.999 power obtained for the ΔE_00_ parameter and TP_00_ parameters of 160 samples were above the desired level. Similarly, the 0.998 power obtained for the CR parameter of 160 samples was above the desired level. The effects of resin groups, mouthwash groups, and measurement times on the TP_00_, CR, and ΔE_00_ are examined in detail in Tables 3, 4 and 5.

**Table 2:** Evaluation of the joint effects of resin groups, mouthwash groups and measurement times (*n* = 160)

	Resin x Mouthwash x Time			
	*F+*	*p*	η2	Post Power
TP_00_	**4702**	0,001 *	**0,227**	**0,999**
CR	**2783**	0,001 *	**0,148**	**0,998**
ΔE_00_	**9201**	0,001 *	**0,365**	**0,999**

*p < 0.05; Mixed Order Analysis of Variance **(F+)**; Effect Size **(η2)**

**Table 3:** Comparison results for TP_00_ measurement

TP_00_		Mouthwash							Test Statistics ^b^	
		Control		Kloroben		ListerineCM		ListerineTC		
T0	Panavia V5	21,39 ± 1,19 ^*j*^		22,69 ± 1,60 ^*j*^		22,45 ± 0,92 ^*j*^		22,14 ± 0,84 ^*hj*^		*F =* 2286 *p =* 0,095 *η*^*2*^ *=* 0,056
	Estecem II	12,90 ± 0,30 ^*k*^		13,47 ± 0,84 ^*k*^		13,33 ± 0,51 ^*k*^		13,20 ± 0,35 ^*k*^		*F =* 0,533 *p =* 0,660 *η*^*2*^ *=* 0,011
	RelyX veneer	13,49 ± 0,37 ^*k*^		13,46 ± 0,56 ^*k*^		14,01 ± 0,57 ^*k*^		13,99 ± 0,36 ^*k*^		*F =* 0,831 *p =* 0,479 *η*^*2*^ *=* 0,017
	NX3	25,01 ± 1,46 ^*b*^		24,65 ± 1,48 ^*b*^		24,90 ± 1,97 ^*b*^		25,17 ± 1,40 ^*b*^		*F =* 0,432 *p =* 0,731 *η*^*2*^ *=* 0,009
Test Statistics ^a^ (*T0-Resin*)		***F =*** **320,588** ***p =*** **0,001** ***η***^***2***^ ***=*** **0,870**		***F =*** **318,078** ***p =*** **0,001** ***η***^***2***^ ***=*** **0,869**		***F =*** **310,020** ***p =*** **0,001** ***η***^***2***^ ***=*** **0,866**		***F =*** **318,313** ***p =*** **0,001** ***η***^***2***^ ***=*** **0,869**		
			ΔTP_00_–1		ΔTP_00_–1		ΔTP_00_–1		ΔTP_00_–1	
T1	Panavia V5	21,80 ± 1,62 ^*j*^	0,411	21,88 ± 0,68 ^*j*^	*−0,806*	22,92 ± 0,78 ^*hj*^	0,474	23,19 ± 1,19 ^*h*^	*1,04*	***F =*** **4319** ***p =*** **0,006** ***η***^***2***^ ***=*** **0,083**
	Estecem II	12,56 ± 0,47 ^*kl*^	− 0,342	12,71 ± 0,85 ^*kl*^	*−0,769*	11,90 ± 1,01 ^*l*^	*− 1428*	13,25 ± 0,29 ^*kl*^	0,05	*F =* 2636 *p =* 0,052 *η*^*2*^ *=* 0,052
	RelyX veneer	13,45 ± 0,50 ^*kl*^	− 0,46	13,35 ± 0,55 ^*kl*^	− 0,112	13,68 ± 0,33 ^*kl*^	− 0,33	13,71 ± 0,35 ^*l*^	− 0,283	*F =* 0,269 *p =* 0,848 *η*^*2*^ *=* 0,006
	NX3	24,14 ± 1,73 ^*b*^	*−0,876*	24,44 ± 1,59 ^*ab*^	−0,211	26,14 ± 1,99 ^*a*^	*1248*	26,29 ± 1,10 ^*a*^	*1121*	***F =*** **10,799** ***p =*** **0,001** ***η***^***2***^ ***=*** **0,184**
Test Statistics ^a^ (*T1-Resin*)		***F =*** **293,084** ***p =*** **0,001** ***η***^***2***^ ***=*** **0,859**		***F =*** **303,599** ***p =*** **0,001** ***η***^***2***^ ***=*** **0,863**		***F =*** **413,905** ***p =*** **0,001** ***η***^***2***^ ***=*** **0,896**		***F =*** **376,58** ***p =*** **0,001** ***η***^***2***^ ***=*** **0,887**		
			ΔTP_00_–7		ΔTP_00_–7		ΔTP_00_–7		ΔTP_00_–7	
T7	Panavia V5	22,20 ± 1,22 ^*hj*^	0,809	22,88 ± 1,65 ^*h*^	0,200	22,81 ± 1,09 ^*hj*^	0,363	22,87 ± 0,88 ^*hj*^	*0,73*	*F =* 0,706 *p =* 0,550 *η*^*2*^ *=* 0,014
	Estecem II	12,40 ± 0,37 ^*kl*^	−0,501	12,75 ± 0,68 ^*kl*^	*−0,723*	12,06 ± 1,24 ^*kl*^	*− 1266*	12,96 ± 0,35 ^*l*^	− 0,244	*F =* 1014 *p =* 0,389 *η*^*2*^ *=* 0,021
	RelyX veneer	13,29 ± 0,38 ^*kl*^	− 0,196	13,47 ± 0,76 ^*kl*^	0,007	11,82 ± 0,6 ^*l*^	*− 2185*	13,64 ± 0,47 ^*k*^	− 0,349	***F =*** **4576** ***p =*** **0,004** ***η***^***2***^ ***=*** **0,087**
	NX3	22,25 ± 2,03 ^*h*^	**− 2765**	23,87 ± 2,34 ^*bh*^	*− 0,766*	25,84 ± 2,08 ^*ab*^	*0,947*	24,71 ± 0,88 ^*ab*^	−0,458	***F =*** **14,980** ***p =*** **0,001** ***η***^***2***^ ***=*** **0,238**
Test Statistics ^a^ (*T7-Resin*)		***F =*** **192,534** ***p =*** **0,001** ***η***^***2***^ ***=*** **0,800**		***F =*** **231,467** ***p =*** **0,001** ***η***^***2***^ ***=*** **0,828**		***F =*** **344,413** ***p =*** **0,001** ***η***^***2***^ ***=*** **0,878**		***F =*** **244,096** ***p =*** **0,001** ***η***^***2***^ ***=*** **0,836**		
Test Statistics ^a^ (*Mouthwash - Panavia V5*)		*F =* 1709 *p =* 0,185 *η*^*2*^ *=* 0,023		***F =*** **5735** ***p =*** **0,004** ***η***^***2***^ ***=*** **0,074**		*F =* 1117 *p =* 0,330 *η*^*2*^ *=* 0,015		*F =* 5417 *p =* 0,005 *η*^*2*^ *=* 0,070		
Test Statistics ^a^ (*Mouthwash - Estecem II)*		*F =* 0,781 *p =* 0,460 *η*^*2*^ *=* 0,011		*F =* 2975 *p =* 0,054 *η*^*2*^ *=* 0,040		***F =*** **10,213** ***p =*** **0,001** ***η***^***2***^ ***=*** **0,125**		***F =*** **0,301** ***p =*** **0,741** ***η***^***2***^ ***=*** **0,004**		
Test Statistics ^a^ (*Mouthwash - RelyX veneer*)		*F =* 0,104 *p =* 0,902 *η*^*2*^ *=* 0,001		*F =* 0,095 *p =* 0,909 *η*^*2*^ *=* 0,001		***F =*** **14,043** ***p =*** **0,001** ***η***^***2***^ ***=*** **0,164**		***F =*** **0,459** ***p =*** **0,633** ***η***^***2***^ ***=*** **0,006**		
Test Statistics ^a^ (*Mouthwash - NX3*)		***F =*** **19,551** ***p =*** **0,001** ***η***^***2***^ ***=*** **0,215**		*F =* 1583 *p =* 0,209 *η*^*2*^ *=* 0,022		***F =*** **7717** ***p =*** **0,001** ***η***^***2***^ ***=*** **0,097**		***F =*** **12,667** ***p =*** **0,001** ***η***^***2***^ ***=*** **0,150**		

F: Mixed Order analysis of variance; Effect Size (***η*2**); ^a^Between-group comparison, ^b^Intra-group comparison, summary statistics are given as mean ± standard deviation. The parts determined in bold are statistically significant (*p* < 0.05). *a > b > j > k > l > h*: Different letters or combinations of letters on the same line represent statistically significant differences (*p* < 0.05)

**Table 4:** Comparison results for CR measurement

CR		Mouthwash				Test Statistics ^b^
		Control	Kloroben	ListerineCM	ListerineTC	
T0	Panavia V5	0,40 ± 0,02 ^*ek*^	0,38 ± 0,03 ^*k*^	0,39 ± 0,01 ^*k*^	0,39 ± 0,01 ^*k*^	*F =* 2106 *p =* 0,102 *η*^*2*^ *=* 0,042
	Estecem II	0,61 ± 0,01 ^*ab*^	0,60 ± 0,02 ^*b*^	0,60 ± 0,02 ^*b*^	0,60 ± 0,01 ^*b*^	*F =* 0,798 *p =* 0,497 *η*^*2*^ *=* 0,016
	RelyX veneer	0,59 ± 0,01 ^*ab*^	0,59 ± 0,02 ^*b*^	0,58 ± 0,01 ^*bc*^	0,58 ± 0,01 ^*bc*^	*F =* 1122 *p =* 0,342 *η*^*2*^ *=* 0,023
	NX3	0,33 ± 0,02 ^*l*^	0,34 ± 0,02 ^*l*^	0,34 ± 0,03 ^*l*^	0,33 ± 0,02 ^*l*^	*F =* 0,380 *p =* 0,768 *η*^*2*^ *=* 0,008
Test Statistics ^a^ (*T0-Resin*)		***F =*** **584,599** ***p =*** **0,001** ***η***^***2***^ ***=*** **0,924**	***F =*** **562,833** ***p =*** **0,001** ***η***^***2***^ ***=*** **0,921**	***F =*** **539,818** ***p =*** **0,001** ***η***^***2***^ ***=*** **0,918**	***F =*** **559,87** ***p =*** **0,001** ***η***^***2***^ ***=*** **0,921**	
T1	Panavia V5	0,40 ± 0,02 ^*e*^	0,40 ± 0,01 ^*e*^	0,38 ± 0,02 ^*f*^	0,37 ± 0,01 ^*f*^	***F =*** **5829** ***p =*** **0,001** ***η***^***2***^ ***=*** **0,108**
	Estecem II	0,62 ± 0,01 ^*ac*^	0,61 ± 0,02 ^*c*^	0,62 ± 0,02 ^*c*^	0,60 ± 0,01 ^*bc*^	*F =* 2364 *p =* 0,074 *η*^*2*^ *=* 0,047
	RelyX veneer	0,59 ± 0,01 ^*ac*^	0,60 ± 0,01 ^*bc*^	0,58 ± 0,01 ^*c*^	0,59 ± 0,01 ^*c*^	*F =* 1155 *p =* 0,329 *η*^*2*^ *=* 0,023
	NX3	0,35 ± 0,03 ^*g*^	0,34 ± 0,02 ^*gl*^	0,32 ± 0,02 ^*l*^	0,32 ± 0,03 ^*l*^	***F =*** **8280** ***p =*** **0,001** ***η***^***2***^ ***=*** **0,147**
Test Statistics ^a^ (*T1-Resin*)		***F =*** **547,021** ***p =*** **0,001** ***η***^***2***^ ***=*** **0,919**	***F =*** **562,575** ***p =*** **0,001** ***η***^***2***^ ***=*** **0,921**	***F =*** **671,384** ***p =*** **0,001** ***η***^***2***^ ***=*** **0,933**	***F =*** **637,044** ***p =*** **0,001** ***η***^***2***^ ***=*** **0,930**	
T7	Panavia V5	0,40 ± 0,02 ^e*k*^	0,38 ± 0,02 ^*k*^	0,38 ± 0,04 ^*ek*^	0,38 ± 0,01 ^*k*^	*F =* 1544 *p =* 0,206 *η*^*2*^ *=* 0,031
	Estecem II	0,62 ± 0,01 ^*a*^	0,61 ± 0,02 ^*ac*^	0,62 ± 0,03 ^*ab*^	0,61 ± 0,01 ^*ab*^	*F =* 0,932 *p =* 0,427 *η*^*2*^ *=* 0,019
	RelyX veneer	0,60 ± 0,01 ^*a*^	0,59 ± 0,02 ^*bc*^	0,59 ± 0,01 ^*b****c***^	0,59 ± 0,01 ^*c*^	***F =*** **0,564** ***p =*** **0,639** ***η***^***2***^ ***=*** **0,012**
	NX3	0,32 ± 0,03 ^*kl*^	0,35 ± 0,04 ^*fkl*^	0,38 ± 0,02 ^*e*^	0,34 ± 0,01 ^*k*^	***F =*** **12,700** ***p =*** **0,001** ***η***^***2***^ ***=*** **0,209**
Test Statistics ^a^ (*T7-Resin*)		***F =*** **376,483** ***p =*** **0,001** ***η***^***2***^ ***=*** **0,887**	***F =*** **17,364** ***p =*** **0,001** ***η***^***2***^ ***=*** **0,897**	***F =*** **493,128** ***p =*** **0,001** ***η***^***2***^ ***=*** **0,911**	***F =*** **432,248** ***p =*** **0,001** ***η***^***2***^ ***=*** **0,900**	
Test Statistics ^a^ (*Mouthwash - Panavia V5*)		*F =* 0,241 *p =* 0,786 *η*^*2*^ *=* 0,003	***F =*** **5825** ***p =*** **0,004** ***η***^***2***^ ***=*** **0,075**	***F =*** **3854** ***p =*** **0,023** ***η***^***2***^ ***=*** **0,051**	***F =*** **6029** ***p =*** **0,003** ***η***^***2***^ ***=*** **0,078**	
Test Statistics ^a^ (*Mouthwash - Estecem II)*		*F =* 2506 *p =* 0,085 *η*^*2*^ *=* 0,034	***F =*** **5149** ***p =*** **0,007** ***η***^***2***^ ***=*** **0,067**	***F =*** **7203** ***p =*** **0,001** ***η***^***2***^ ***=*** **0,092**	*F =* 1488 *p =* 0,229 *η*^*2*^ *=* 0,020	
Test Statistics ^a^ (*Mouthwash - RelyX veneer*)		*F =* 0,136 *p =* 0,873 *η*^*2*^ *=* 0,002	*F =* 0,577 *p =* 0,563 *η*^*2*^ *=* 0,008	*F =* 0,225 *p =* 0,799 *η*^*2*^ *=* 0,003	*F =* 1338 *p =* 0,266 *η*^*2*^ *=* 0,018	
Test Statistics ^a^ (*Mouthwash - NX3*)		***F =*** **19,287** ***p =*** **0,001** ***η***^***2***^ ***=*** **0,212**	*F =* 1357 *p =* 0,261 *η*^*2*^ *=* 0,019	***F =*** **6789** ***p =*** **0,002** ***η***^***2***^ ***=*** **0,087**	***F =*** **8399** ***p =*** **0,001** ***η***^***2***^ ***=*** **0,105**	

F: Mixed Order analysis of variance; Effect Size (**η2**); ^a^Between-group comparison, ^b^Intra-group comparison, summary statistics are given as mean ± standard deviation. The parts determined in bold are statistically significant (*p* < 0.05). *a > b > c > e > f > g > k > l*: Different letters or combinations of letters on the same line represent a statistically significant difference (*p* < 0.05)

**Table 5:** Comparison results for ΔE_00_ measurement

ΔE_00_		Mouthwash				Test Statistics ^b^
		Control	Kloroben	ListerineCM	ListerineTC	
T1	Panavia V5	1,94 ± 0,68 ^*h*^	2,22 ± 0,55 ^*h*^	4,65 ± 0,52 ^*c*^	2,64 ± 0,64 ^*gh*^	***F =*** **38,825** ***p*** **= 0,001** ***η***^***2***^ ***=*** **0,447**
	Estecem II	1,10 ± 0,29 ^*k*^	0,92 ± 0,27 ^*m*^	5,71 ± 2,07 ^*a*^	1,07 ± 0,41 ^*l*^	***F =*** **195,288 p = 0,001** ***η***^***2***^ ***=*** **0,803**
	RelyX veneer	1,42 ± 0,49 ^*k*^	0,61 ± 0,25 ^*m*^	1,97 ± 0,62 ^*k*^	1,08 ± 0,32 ^*kl*^	***F =*** **10,926 p = 0,001** ***η***^***2***^ ***=*** **0,185**
	NX3	1,79 ± 0,33 ^*hk*^	0,46 ± 0,29 ^*m*^	3,78 ± 0,82 ^*e*^	3,66 ± 0,30 ^*e*^	***F =*** **28,601 p = 0,001** ***η***^***2***^ ***=*** **0,373**
Test Statistics ^a^ (*T1-Resin*)		***F =*** **3849** ***p =*** **0,001** ***η***^***2***^ ***=*** **0,074**	***F =*** **25,173** ***p =*** **0,001** ***η***^***2***^ ***=*** **0,344**	***F =*** **91,847** ***p =*** **0,001** ***η***^***2***^ ***=*** **0,657**	***F =*** **44,17** ***p =*** **0,001** ***η***^***2***^ ***=*** **0,479**	
T7	Panavia V5	3,29 ± 0,67 ^*g*^	2,46 ± 0,68 ^*g*^	6,05 ± 0,76 ^*ac*^	3,29 ± 0,91 ^*g*^	***F =*** **43,335** ***p =*** **0,001** ***η***^***2***^ ***=*** **0,474**
	Estecem II	1,55 ± 0,33 ^*jk*^	1,48 ± 0,90 ^*jk*^	6,37 ± 1,41 ^*ac*^	1,94 ± 0,36 ^*hj*^	***F =*** **67,36** ***p =*** **0,001** ***η***^***2***^ ***=*** **0,584**
	RelyX veneer	1,08 ± 0,39 ^*k*^	0,61 ± 0,25 ^*m*^	2,65 ± 0,32 ^*h*^	1,77 ± 0,40 ^*h*^	***F =*** **12,712** ***p =*** **0,001** ***η***^***2***^ ***=*** **0,209**
	NX3	2,08 ± 0,51 ^*hk*^	2,86 ± 0,66 ^*gh*^	5,09 ± 0,88 ^*cd*^	4,48 ± 0,68 ^*d*^	***F =*** **31,699** ***p =*** **0,001** ***η***^***2***^ ***=*** **0,398**
Test Statistics ^a^ (*T7-Resin*)		***F =*** **14,693** ***p =*** **0,001** ***η***^***2***^ ***=*** **0,234**	***F =*** **15,272** ***p =*** **0,001** ***η***^***2***^ ***=*** **0,241**	***F =*** **38,289** ***p =*** **0,001** ***η***^***2***^ ***=*** **0,444**	***F =*** **26,267** ***p =*** **0,001** ***η***^***2***^ ***=*** **0,354**	
Test Statistics ^a^ (*Mouthwash - Panavia V5*)		***F =*** **40,170** ***p =*** **0,001** ***η***^***2***^ ***=*** **0,218**	***F =*** **4292** ***p =*** **0,040** ***η***^***2***^ ***=*** **0,029**	*F =* 2473 *p =* 0,118 *η*^*2*^ *=* 0,017	*F =* 1817 *p =* 0,180 *η*^*2*^ *=* 0,012	
Test Statistics ^a^ (*Mouthwash - Estecem II)*		*F =* 1228 *p =* 0,270 *η*^*2*^ *=* 0,008	***F =*** **6779** ***p =*** **0,010** ***η***^***2***^ ***=*** **0,045**	*F =* 0,461 *p =* 0,498 *η*^*2*^ *=* 0,003	***F =*** **10,825** ***p =*** **0,001** ***η***^***2***^ ***=*** **0,070**	
Test Statistics ^a^ (*Mouthwash - RelyX veneer*)		*F =* 2473 *p =* 0,118 *η*^*2*^ *=* 0,017	*F =* 0,461 *p =* 0,498 *η*^*2*^ *=* 0,003	***F =*** **9962** ***p =*** **0,002** ***η***^***2***^ ***=*** **0,065**	***F =*** **10,280** ***p =*** **0,002** ***η***^***2***^ ***=*** **0,067**	
Test Statistics ^a^ (*Mouthwash - NX3*)		*F =* 1817 *p =* 0,180 *η*^*2*^ *=* 0,012	***F =*** **10,825** ***p =*** **0,001** ***η***^***2***^ ***=*** **0,070**	***F =*** **37,713** ***p =*** **0,001** ***η***^***2***^ ***=*** **0,208**	***F =*** **14,642** ***p =*** **0,001** ***η***^***2***^ ***=*** **0,092**	

F: Mixed Order analysis of variance; Effect Size (**η2**); ^a^Between-group comparison, ^b^Intra-group comparison, summary statistics are given as mean ± standard deviation. The parts determined in bold are statistically significant (*p* < 0.05). *a > c > d > e > g > h > j > k > l > m*: Different letters or combinations of letters on the same line represent a statistically significant difference (*p* < 0.05)

### TP_00_ parameter results (Fig. 1)

While the measurement taken at T1 time in the Kloroben (Drogsan) group had a lower TP_00_ average than the measurements taken at T0 and T7 times, the measurement taken at T1 time in the Listerine TC (Johnson & Johnson) group had a higher TP_00_ mean than the measurements taken at T0 and T7 times. The mean of the Control group at T0 time was lower than that of the Kloroben (Drogsan) group. At T1, the mean of the Control and Kloroben (Drogsan) groups was lower than that of the Listerine TC (Johnson & Johnson) group (*F* = 2.847; *F* = 4.319) (Table 3).

**Fig. 1:**
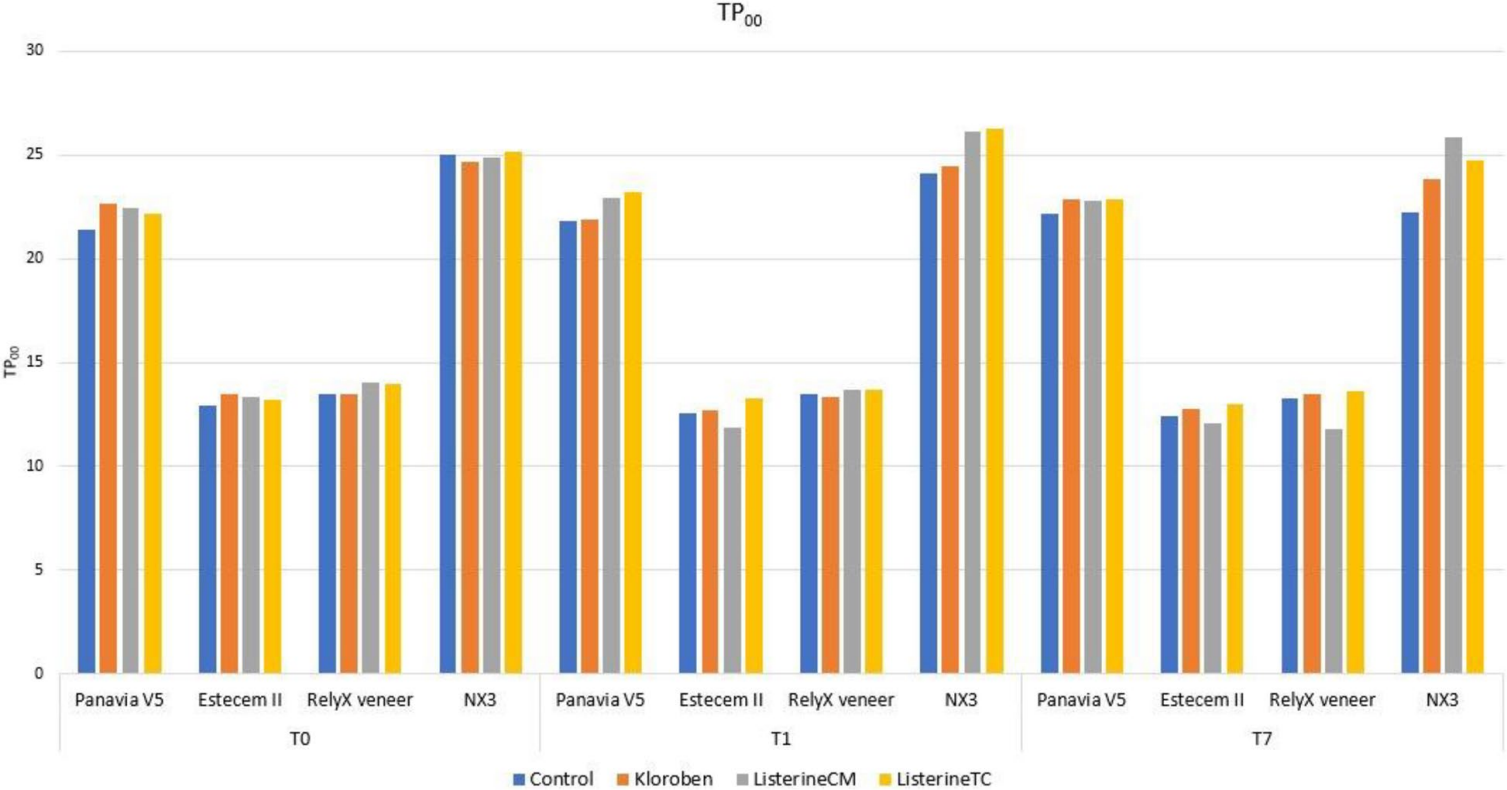
Graphical representation of TP_00_ parameters

In the Estecem II (Tokuyama) and RelyX veneer (3 M ESPE) groups, the Listerine CM (Johnson & Johnson) group showed statistically significant differences at all three measurement times (F* = 10.213, F* = 14.043). There was no significant difference in the measurement times in terms of the mouthwash groups (*p* > 0.05) (Table 3).

In the NX3 (Kerr) group, the control, Listerine CM (Johnson & Johnson), and Listerine TC (Johnson & Johnson) groups differed significantly statistically at all three measurement times (F* = 19.551; F* = 7.717; F* = 12.667) (Table 3).

The translucency change value (ΔTP_00_) was observed to be above acceptable limits in the T7 time period in the NX3 (Kerr) group in control. In addition, ΔTP_00_ were calculated over multiple time periods in the NX3 (Kerr) group and was shown to be above perceptible limits in the mouthwash group. ΔTP_00_ was observed to be above the perceptible limit in the Panavia V5 (Kuraray Noritake) group in the Kloroben (Drogsan) and Listerine TC (Johnson & Johnson) groups in the T1 time period and in the control and Listerine TC (Johnson & Johnson) groups in the T7 time period. In the Estecem II (Tokuyama) group, ΔTP_00_ were above perceptible limits with regard to the Kloroben (Drogsan) and Listerine CM (Johnson & Johnson) groups in both time periods. In the Rely X veneer (3 M ESPE) group, a result above the perceptible limit was observed only in the Listerine CM (Johnson & Johnson) group at the T7 time period (Table 3).

### CR parameter results (Fig. 2)

In the Panavia V5 (Kuraray Noritake) group, there was a statistically difference between mouthwashes only in the T1 time. At T1 time, the mean of the Control and Kloroben (Drogsan) groups were higher than those of the Listerine CM (Johnson & Johnson) and Listerine TC (Johnson & Johnson) groups (*F* = 5.829) (Table 4).

**Fig. 2:**
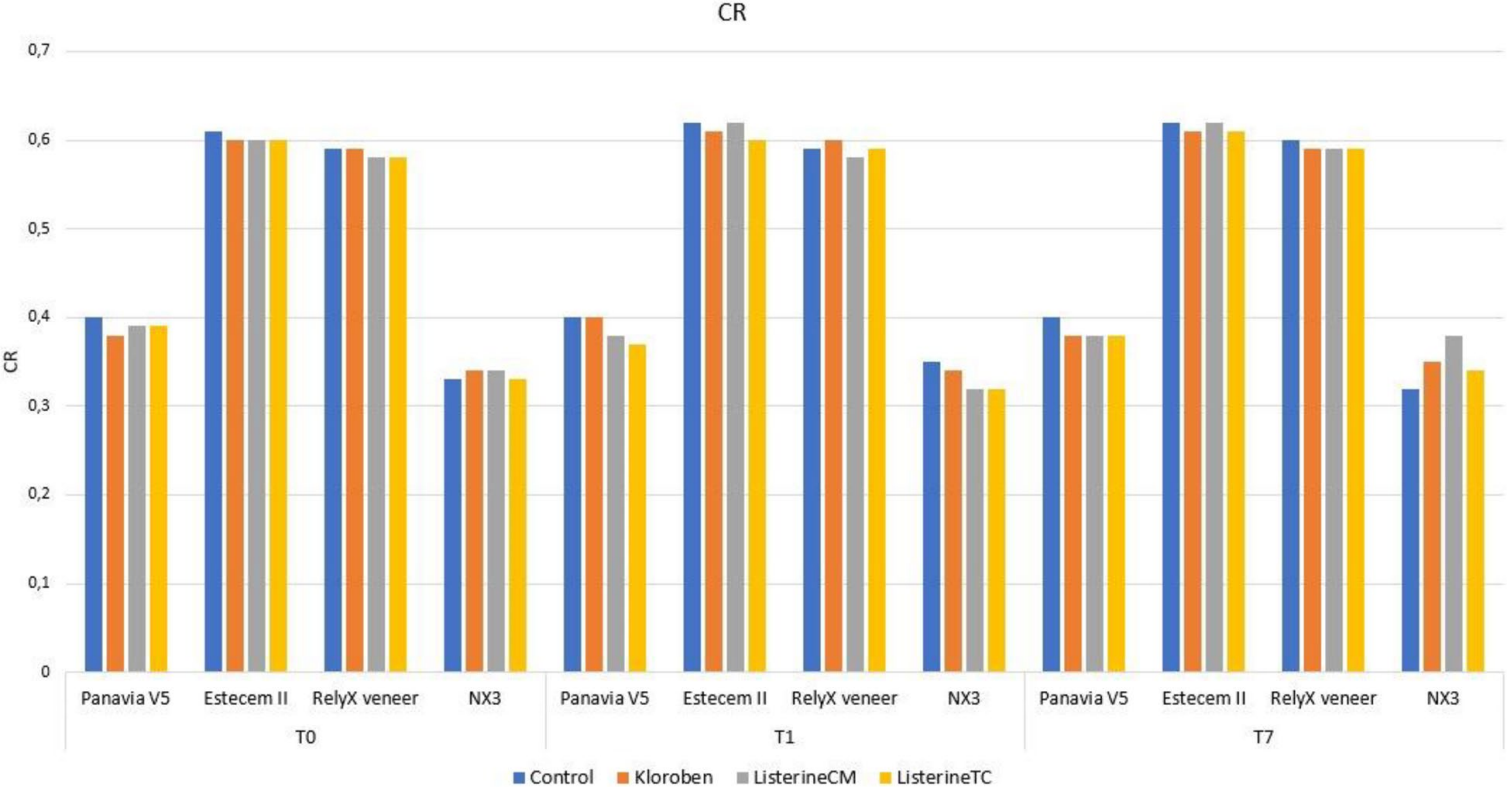
Graphical representation of CR parameters

Kloroben (Drogsan) and Listerine CM (Johnson & Johnson) groups in the Estecem II (Tokuyama) group showed statistically significant differences in CR parameter at the three measurement times (F* = 5.149; F* = 7.203).

In the RelyX veneer (3 M ESPE) group, the means of the mouthwash groups did not differ significantly statistically at the three measurement times (*p* > 0.05) (Table 4).

In the NX3 (Kerr) group, the Control, Listerine TC (Johnson & Johnson), and Listerine CM (Johnson & Johnson) groups differed significantly statistically at the three measurement times (F* = 19.287; F* = 6.789; F* = 8.399) (Table 4).

### ΔE_00_ parameter results (Fig. 3)

In all resin groups, ΔE_00_ values at T1 and T7 showed significant differences in different mouthwashes (*p* = 0.001).

**Fig. 3:**
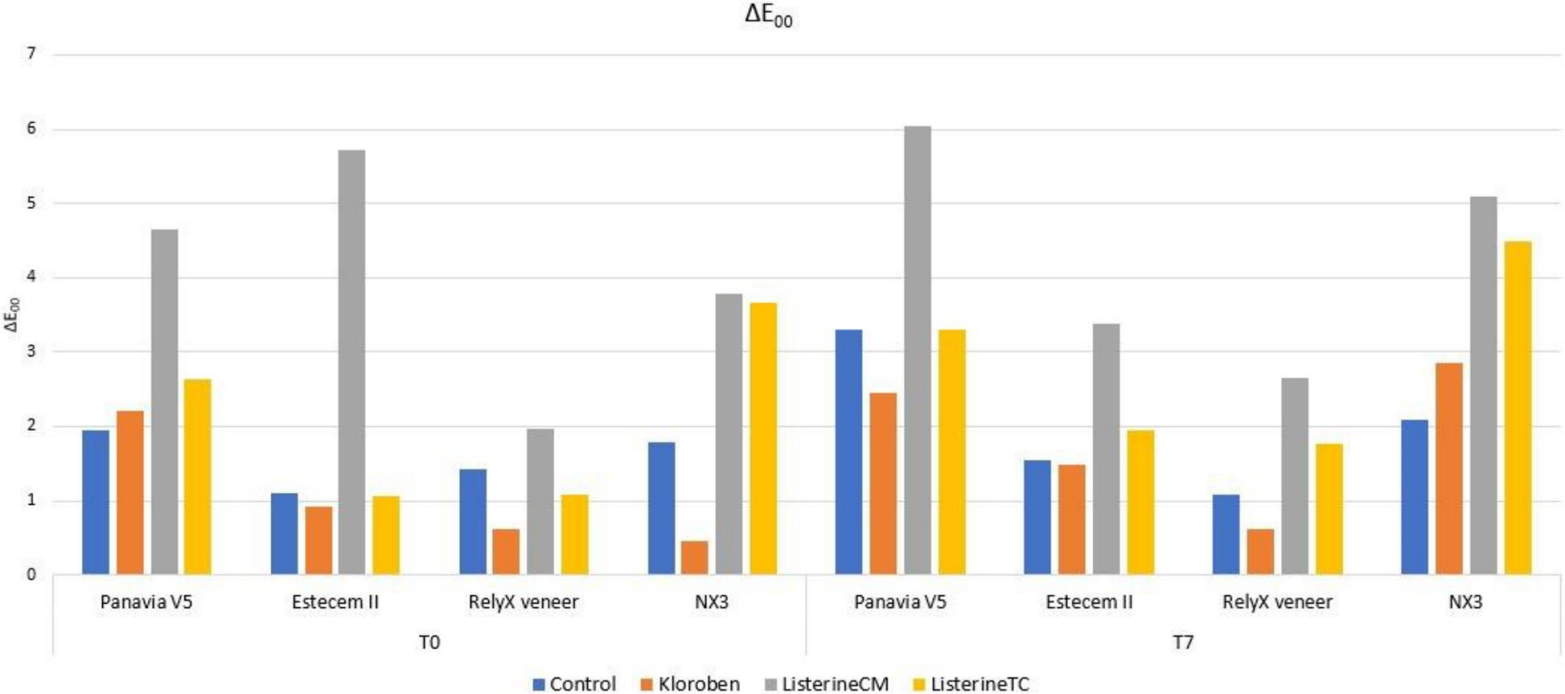
Graphical representation of ΔE_00_ parameter

In the Panavia V5 (Kuraray Noritake) group, the ΔE_00_ value was above acceptable limits in all time periods and in all mouthwash groups (Table 5).

In the Estecem II (Tokuyama) group, the means of Listerine CM (Johnson & Johnson) was above acceptable limits in both time periods and Listerine TC (Johnson & Johnson) was above acceptable limits in the T7 time period. All other data were above perceptible limits (Table 5).

While the ΔE_00_ value in the RelyX veneer (3 M ESPE) group was below the perceptible/acceptable value in Kloroben (Drogsan) in both time periods, it was above the acceptable value in the case of Listerine CM (Johnson & Johnson). It was above the perceptible limit in all other mouthwashes at all time periods (Table 5).

In the NX3 (Kerr) group, the ΔE_00_ value was above acceptable limits for all mouthwashes in the T7 time period. In the T1 time period, it was above acceptable limits in the Listerine CM (Johnson & Johnson) and Listerine TC (Johnson & Johnson) groups, while it was below the acceptable limits in the other groups (Table 5).

## Discussion

This study consists of 4 resin cement, 3 mouthwash groups, and 1 control group. The effect of mouthwash on the colouration and optical properties of resin cement was evaluated in the present study. At the end of the 1st and 7th-day evaluations, the changes in TP_00_, CR, and ΔE_00_ parameters are statistically significant. Based on the results of the present study, the initial hypothesis tested was rejected.

Turgut et al. [25] reported that, due to the high colour stability of ceramic materials resin cement is the principal cause of colour change in restorative treatments with ceramic laminate veneers. Therefore, in this study, laminate veneers were not produced. Resin discs were created to evaluate the optical properties of resin cement.

Since it was not possible to acquire a suitable fluorimeter for this study, opalescence/fluorescence could not be evaluated.

The CIEDE 2000 difference formula was developed to eliminate the deficiencies of the CIELAB(L*a*b) colour system [22]. Researchers have reported that the CIEDE 2000 difference formula provides more accurate and appropriate results within the acceptable and perceptible range [23]. Therefore, the CIEDE 2000 difference formula was used in this study. In this study, as stated by Paravina et al. [21], the clinically perceptible/ acceptable limit was accepted as ΔE_00_ = 0.8/1.8.

The pH values, active ingredients, and alcohol concentrations of mouthwashes can have detrimental effects on the restorative material. Many colour stabilization studies have reported that the colour stability of resin materials depends on the resin matrix and filler compositions [25–27].

In the present study, ΔE_00_ increased over time in all groups. Among all groups, at the end of the 1st and 7th days, the greatest colour changes in all resin cement groups occurred in case of Listerine CM (Johnson & Johnson). This value was above clinically acceptable limits. Listerine TC (Johnson & Johnson) exhibited a colour change above the acceptable limit in Panavia V5 (Kuraray Noritake), in NX3 (Kerr) groups at T1 and T7 times and in the Estecem II (Tokuyama) group at T7 time. Additionally, Listerine TC (Johnson & Johnson) caused changes beyond perceptible limit in the RelyX veneer (3 M ESPE) group. Based on these results, it can be stated that the use of Listerine CM (Johnson & Johnson) and Listerine TC (Johnson & Johnson) caused colour changes in resin-based materials. The reason for this may be related to the chemical content of the mouthwash, and also the content of resin cement. BISGMA and TEGDMA found in Panavia V5 (Kuraray Noritake) are monomers with an affinity for water, but resin cements other than NX3 (Kerr) used in the study contain these monomers. The difference in Panavia V5 (Kuraray Noritake) may be related to the type, content, and proportions of fillers. In addition, the colour changes of resin cement are affected by the oxidation of the resin matrix and amine triggers or aliphatic. NX3 (Kerr) contains a tertiary amine. In addition, unreacted benzoyl peroxide which it contains may have caused discolouration over time. Some researchers [28, 29] have concluded that lighter shades of resin-based materials exhibit higher discolouration after a period of soaking. In this study, NX3 (Kerr) was in clear colour, that is, the clearest and translucent colour. This may be another reason for the colouration in the NX3 (Kerr).

In the present study, colour change values were found on the 7th day compared to the 1st day in both the control group and mouthwash groups. The residence time in solutions is quite effective in terms of the polymer chains of resin materials. Significant adverse effects occur with longer exposure to the solution [30]. This supports the fact that resin-based materials kept in solutions for 7 days exhibited more water absorption than on the 1st day of our study. Similarly, Von Fraunhofer et al. [31] reported that the absorption of the liquid increased with long-term presence in the mouthwash. Panavia V5 (Kuraray Noritake) has two monomers that have an affinity for water: BISGMA and TEGDMA. Depending on this, in the control group, the colour change in case of Panavia V5 may have been greater than was the case in the other resin cements.

It is reported that alcohol is also a good dimethacrylate solvent, one which affects the mechanical properties of resin-based materials, and increases water absorption and solubility [32]. In resin-based materials, this can affect water absorption and solubility by softening the polymeric matrix and increasing the number of unreacted monomers and oligomers [33]. Weiner et al. [34] reported that water absorption increases in alcoholic mouthwashes compared to non-alcoholic mouthwashes. Organic solutions such as ethanol have a solubility parameter close to that of BisGMA [35]. Maximum liquid uptake occurs when the solubility parameter of the liquid is close to that of the polymer [36]. Sarrett et al. [37] reported that beverages containing at least 9% ethanol will increase the degradation of composite resins by causing water absorption and matrix swelling, depending on the hydrophilicity of the polymer matrix and the localization of hydrolysable groups on the matrix chains. This can be explained by the fact that the Listerine CM (Johnson & Johnson) used in our study causes a colour change in all materials. Listerine TC (Johnson & Johnson), on the other hand, did not cause colour change as much as Listerine CM (Johnson & Johnson) in all materials. It caused perceptible staining beyond, although within acceptable limits, in Estecem II (Tokuyama) and RelyX veneer (3 M ESPE). This situation can be explained by the difference in the content, pH, and alcohol ratio of the mouthwashes.

Mouthwashes can contain water, antimicrobial agents, salts, preservatives, and sometimes alcohol in their makeup and have different pHs according to the differences in their concentrations; In addition to preventing caries, they are widely used for reducing bad breath and providing fresh breath [38]. However, depending on their pH, active substances, ingredients, and alcohol concentration, they can have harmful effects on teeth and restorative materials. Alcohol concentration and low pH value of mouthwashes play a key role in this mechanism [18]. Listerine CM (Johnson & Johnson) (pH = 3.98) and Listerine TC (Johnson & Johnson) (pH = 3.43) mouthwash have low pH. Low-pH solutions produce methacrylic acid, which causes enzymatic degradation over time [39]. It also causes low pH in the acid resin matrix, catalyses the hydrolysis of ester groups from dimethacrylate monomers to form carboxylic acid and alcohol molecules, and can promote monomer separation [40, 41]. Since it has been reported that the polymer matrix is more affected in the presence of acid and significant negative effects occur even in the presence of a small amount of diluting monomer such as TEGDMA [42], this may explain the colouration of the TEGDMA-containing resin cements in our study. RelyX veneer (3 M ESPE), on the other hand, was highly resistant to colouration except with regard to Listerine CM (Johnson & Johnson). This can be explained by the ratio of RelyX veneer (3 M ESPE) filler types, and amounts.

Kloroben (Drogsan) caused a colour change at the end of the 7th day. Kloroben (Drogsan) also caused colouration in Panavia V5 (Kuraray Noritake) and NX3 (Kerr). Based on these results, these effects are related to both mouthwash and material content, while colour changes in Listerine CM (Johnson & Johnson) in all groups are related to Listerine CM’s (Johnson & Johnson) contents. In addition, RelyX veneer (3 M ESPE) has shown resistance in other mouthwashes with the exception of Listerine CM (Johnson & Johnson). We think that this may be due to the different monomer structures of composite resins, filler particles, and differences in the content and pH of the mouthwashes used.

It has been reported in the literature that translucency is affected by many factors. Azzopardi et al. [43] reported that organic matrix and filler particles can affect the translucency of experimental composites. It has also been reported in studies that the translucency of restorative materials is dependent on absorption and scattering [8]. Although scattering in composite resins occurs due to the refractive index mismatch between the organic matrix and the filler particles, and the size and distribution of inorganic fillers, the absorption is produced by the organic matrix (monomer matrix reactivity) [44].

In their review, Salas et al. [24] reported that the perceptibility threshold of the translucency parameter for CIEDE2000 was 0.62 and the acceptability threshold was 2.62. In this study, these values were accepted as threshold values.

In the present study, Listerine CM (Johnson & Johnson) decreased TP_00_ parameters in case of Estecem II (Tokuyama), RelyX veneer (3 M ESPE), and NX3 (Kerr) groups. This may be related to mouthwash. On the other hand, NX3 (Kerr) also had decreased TP_00_ parameters in the control and Listerine TC (Johnson & Johnson) groups. In the literature, studies showed that the composition of the matrix and the filler, the refractive index difference between the inorganic filler particles and the matrix phase, the size of the fillers, and the particle size ratio, all affect the optical properties of the resin-based materials [45]. ΔTP_00_ was above acceptable limits in the T7 time period only in the NX3 group. Therefore, this result wth regard to NX3 (Kerr) may be related to the material used. However, there was no significant difference in Listerine CM (Johnson & Johnson) in Panavia V5 (Kuraray Noritake). Kloroben (Drogsan) and Listerine TC (Johnson & Johnson) values decreased in TP_00_. It has been stated that the percentage of Bis-GMA contained in resin-based materials significantly affects the translucency of resin-based materials containing silica as a filler [43]. The filler size, shape, and content of the Bis-GMA-based materials used in our study are different as is the amount of Bis-GMA they contain. The refractive index of Bis-GMA is close to the refractive index of silica filler. The difference in TP_00_ values may also be due to the different resin matrices and filler size, amount, and distribution of the materials. The translucency values of resin-based materials can also be affected by the degree of polymerization. The increase in the polymerization of the resin matrix and the change in the refractive index can also change the degree of translucency. The increase in polymerization further increases the difference in refractive index between the resin matrix and the inorganic filler [46].

Contrast ratio values, another parameter whose translucency is evaluated, did not change in the case of RelyX veneer (3 M ESPE). However, the values increased at the end of 1 day in Panavia V5 (Kuraray Noritake), and became opaque and approached the initial values at the end of 7 days. Cr values increased in Kloroben (Drogsan) and Listerine CM in Estecem II (Tokuyama). On the other hand, while NX3 (Kerr) became opaque in the control, the CR values increased in Listerine TC (Johnson & Johnson) and decreased in Listerine CM (Johnson & Johnson). While Estecem II (Tokuyama) became opaque to the greatest extent in Kloroben (Drogsan) and Listerine CM (Johnson & Johnson), the difference in Estecem II (Tokuyama) was not significant in Listerine TC (Johnson & Johnson). In Listerine TC (Johnson & Johnson), NX3 (Kerr) became opaque, while in Panavia V5 (Kuraray Noritake) it became opaque at the end of 1 day, and values approached the initial values at the end of the 7th day. The birefringent nature of crystals and light scattering at grain boundaries, as well as grain size, pores, the presence of second-phase particles, and surface roughness, have all been linked to the optical properties and the process of light scattering in polycrystalline systems [47]. The differences between the materials can be explained by these.

### Limitations

This study has several limitations when compared to clinical investigations because it was created and carried out in vitro. In reality, neither the aging effects of environmental variables on resin-based materials nor the effects of saliva in actual clinical practice were taken into account. The resin-based materials are not continuously exposed to the solutions in clinical conditions. Alternating times of saliva exposure with periods when the restoration is exposed to mouthwashes allows the saliva to adjust the pH and/or other environmental changes. Resin cements are not directly exposed to the oral environment with all their surfaces, as they all adhere to ceramic on the tooth. As a result, resin cements will only be exposed to mouthwash in terms of the marginal portion of the restoration. However, in this study, all of the resin cement was affected by the mouthwash. It should be taken into account that this might well increase change in colour tone [48]. Furthermore, additional in vitro and in vivo studies are needed to arrive at more accurate conclusions with regard to the materials tested.

## Conclusions

Within the limitations of this in vitro study, the following conclusions can be drawn;

1. The colour stability of resin cement depends on both the resin cement content and the mouthwash used.
2. Exposure time to mouthwashes increases the colour stability of the resin cement.
3. The translucency of the resin cement depends on both the resin cement content and the mouthwash used.
4. Exposure time to mouthwashes reduce the translucency of the resin cement.
5. Clinicians should consider that the colour of laminate veneers will be affected by the content of the resin cement used and the use of mouthwash on the part of the patient.

## Data Availability

The datasets used and/or analysed during the current study available from the corresponding author on reasonable request.
